# Molecular Systematics of the Firefly Genus *Luciola* (Coleoptera: Lampyridae: Luciolinae) with the Description of a New Species from Singapore

**DOI:** 10.3390/ani11030687

**Published:** 2021-03-04

**Authors:** Wan F. A. Jusoh, Lesley Ballantyne, Su Hooi Chan, Tuan Wah Wong, Darren Yeo, B. Nada, Kin Onn Chan

**Affiliations:** 1 Lee Kong Chian Natural History Museum, National University of Singapore, Singapore 117377, Singapore; 2 School of Agricultural and Wine Sciences, Charles Sturt University, Wagga Wagga 2678, Australia; lballantyne@csu.edu.au; 3 Central Nature Reserve, National Parks Board, Singapore 573858, Singapore; chan_su_hooi@nparks.gov.sg; 4 National Parks Board HQ (Raffles Building), Singapore Botanic Gardens, Singapore 259569, Singapore; tuanwah@gmail.com; 5 Department of Biological Sciences, National University of Singapore, Singapore 117543, Singapore; dbsdy@nus.edu.sg; 6 Forest Biodiversity Division, Forest Research Institute Malaysia, Kepong 52109, Malaysia; nada@frim.gov.my

**Keywords:** *Hotaria*, *Luciola cruciata*, *Luciola owadai*, Nee Soon Swamp Forest, taxonomy, phylogenetics, conservation

## Abstract

**Simple Summary:**

Fireflies have a scattered distribution in Singapore but are not as uncommon as many would generally assume. A nationwide survey of fireflies in 2009 across Singapore documented 11 species, including “*Luciola* sp. 2”, which is particularly noteworthy because the specimens were collected from a freshwater swamp forest in the central catchment area of Singapore and did not fit the descriptions of any known *Luciola* species. Ten years later, we revisited the same locality to collect new specimens and genetic material of *Luciola* sp. 2. Subsequently, the mitochondrial genome of that population was sequenced and specimens were subjected to rigorous morphological examinations. We then collated published mitogenomes and shorter mitochondrial markers from closely related taxa to infer a phylogeny for the subfamily Luciolinae. Our results reveal that *Luciola* sp. 2 is both genetically and morphologically distinct from other congeners and is thus described herein as a new species *Luciola singapura* sp. nov. This marks the first time since 1909 that a new species of luminous firefly has been discovered in Singapore, highlighting the need for continued biodiversity research, even in small, well-studied and highly developed countries such as Singapore that can still harbor undescribed biodiversity.

**Abstract:**

The firefly genus *Luciola* sensu McDermott contains 282 species that are distributed across major parts of Asia, Europe, Africa, Australia, and the Pacific islands. Due to phenotypic similarities, species identification using external morphological characters can be unreliable for this group. Consequently, decades of piecemeal taxonomic treatments have resulted in numerous erroneous and contentious classifications. Furthermore, our understanding of the group’s evolutionary history is limited due to the lack of a robust phylogenetic framework that has also impeded efforts to stabilize its taxonomy. Here, we constructed molecular phylogenies of *Luciola* and its allies based on combined mitogenomes and *Cytochrome c oxidase subunit 1* (*COX1*) sequences including a newly sequenced mitogenome of an unidentified taxon from Singapore. Our results showed that this taxon represents a distinct and hitherto undescribed evolutionary lineage that forms a clade with *L. filiformis* from Japan and *L. curtithorax* from China. Additionally, the Singaporean lineage can be differentiated from other congeners through several external and internal diagnostic morphological characters, and is thus described herein as a new species. Our phylogeny also strongly supported the paraphyly of *Luciola* with regard to *L. cruciata* and *L. owadai*, which were inferred to be more closely related to the genus *Aquatica* as opposed to other members of *Luciola* sensu stricto. The genus *Hotaria* was inferred as a derived clade within *Luciola* (sister to *L. italica*), supporting its status as a subgenus of *Luciola* instead of a distinct genus. This is the first time since 1909 that a new species of luminous firefly has been discovered in Singapore, highlighting the need for continued biodiversity research, even in small, well-studied and highly developed countries, such as Singapore.

## 1. Introduction

Firefly beetles (Coleoptera: Lampyridae) comprise over 2000 species globally. The subfamily Luciolinae—all of which are exclusively flashing fireflies—represents a diverse subfamily (>400 species) distributed across Europe, Africa, Asia, Australia, and the Pacific islands [1,2]. The bulk of lucioline species (~303 species) occur in Southeast (SE) Asia and the Australopacific region, which encompasses the following geographic units: India, Sri Lanka, Bangladesh, China (including Hong Kong), Taiwan, Korea, Japan, Myanmar, Laos, Cambodia, Vietnam, Thailand, Malaysia, Singapore, Indonesia, Philippines, the Republic of Palau, the Federated States of Micronesia, Papua New Guinea, West Papua, Solomon Islands, New Caledonia, Vanuatu, Fiji, and Australia [3]. The continents of Africa and Europe have a combined total of at least 100 lucioline species [4].

In the 19th century, the taxonomy of Luciolinae relied heavily on external features such as colour pattern of the dorsal surface, male terminal abdomen, and prominence of the elytral carina as diagnostic characters [2,5]. However, for the genus *Luciola* Laporte, the most speciose genus in the subfamily Luciolinae [4,6,7], species identification based on external morphological characters is problematic due to overlapping phenotypic similarities [2,8]. Decades of piecemeal taxonomic treatments coupled with the lack of a robust phylogenetic framework have resulted in the genus being a dumping ground for ambiguous taxa, leading to protracted taxonomic instability, contentious classifications, and unnatural taxonomic groupings [9,10,11].

In 1966, 282 species were listed under the genus *Luciola* by McDermott in the world catalogue of Lampyridae, to which 279 species were assigned to the subgenus *Luciola*, and one species, respectively, to the following subgenera: *Hotaria* Yuasa, *Photuroluciola* Pic, and *Pygoluciola* Wittmer [4]. After McDermott, a large number of SE Asian and Australopacific lucioline taxa (mostly within the subgenus *Luciola*) were subjected to numerous revisions, redefinitions, and rearrangements, leading to widespread taxonomic changes and instability [1,2,3,5,8,10,12,13,14,15]. 

In 2019, Ballantyne et al. [3] assigned 17 species to *Luciola* s. str.—a group defined by several morphological characteristics shared with the type species of *Luciola, L. italica* (L. [16]), primarily: 1. visible view of aedeagal lateral lobes (LL) beside the medium lobe (ML), with a clear separation of the aedeagal lateral lobe along the inner dorsal length; 2. presence of either curved or arched medium lobe terminating in a preapical ventral point; 3. presence of elongated, narrow, pointed lobes (“leaf like”) arising from the inner ventral margins of the lateral lobes (some species, including *L. italica*) [8]. The remaining 40 species in the genus *Luciola* have yet to undergo thorough morphological assessments. Of these, Ballantyne et al. [3] highlighted seven species (including *Luciola costata* Pic) that could potentially be transferred to the genus *Curtos* without formalizing the move; the remaining 33 species (including two synonyms) were assigned to *Luciola* s. lato [3] which is a loose category that lacks a proper definition.

Two well-known and revered firefly species from Japan, *Luciola cruciata* Motschulsky and *Luciola owadai* Sâtô and Kimura do not fit the definition of *Luciola* and were provisionally considered as “taxonomic position unresolved” [3]. Their distinction from *Luciola* s. str. was further corroborated by molecular phylogenetic analyses [15]. Meanwhile, *Photuroluciola* and *Pygoluciola* were removed from the subgenus *Luciola* sensu McDermott and elevated to the genus rank [12,13,17,18,19], while *Luciola parvula* Kiesenwetter, the type species of the subgenus *Hotaria* sensu McDermott was transferred to *Luciola* s. str. [3,15]. Nonetheless, the taxonomic rank of *Hotaria* is still in question because after McDermott, *Hotaria* was still being recognized as a distinct genus in some studies, e.g., [20,21], while most studies recognized it as a subgenus of *Luciola,* e.g., [22]. *Hotaria* previously consisted of four species: *Luciola parvula, L. unmunsana* Doi, *L. papariensis* Doi, and *L. tsushimana* Nakane; the latter two were subsequently synonymised with *L. unmunsana* [23].

In Singapore, a small, highly urbanized, and bustling city-state in SE Asia, fireflies have a scattered distribution but are not as uncommon as many would generally assume. A nationwide survey of fireflies in 2009 at 14 sites across Singapore documented 11 species [24]. Included in that survey was an unidentified species, “*Luciola* sp. 2”, which was particularly noteworthy because the specimens were collected from a freshwater swamp forest in the central catchment area of Singapore and did not fit the descriptions of any known *Luciola* species. Several years later, while examining the Lampyridae collection in the Zoological Reference Collection (ZRC) at the Lee Kong Chian Natural History Museum in Singapore, the first author discovered three additional dry specimens collected in 1989 and 1990 from the same locality that were morphologically similar to *Luciola* sp. 2. For this study, we revisited the same locality to collect new specimens and genetic material of *Luciola* sp. 2. Subsequently, the mitochondrial genome of that population was sequenced and specimens were subjected to rigorous morphological examinations. We then collated published mitogenomes from closely related taxa to infer a molecular phylogeny to serve as an evolutionary framework to aid taxonomic classification and species delineation. The mitogenomic dataset was also supplemented with shorter mitochondrial markers from additional taxa to fill-in taxonomic sampling gaps. Results from morphological examinations and phylogenetic analyses were used to determine if (1) *Luciola* sp. 2 represents a unique evolutionary lineage warranting specific recognition and (2) the morphological definition of *Luciola* s. str. is consistent within an evolutionary framework that is supported by robust molecular data.

After 30 years since its initial discovery, our results indicate that *Luciola* sp. 2 is a distinct evolutionary lineage that is both genetically and morphologically distinct from other congeners and is thus described herein as a new species, *Luciola singapura* sp. nov. This marks the first time since 1909 that a new species of luminous firefly has been discovered in Singapore [25], highlighting the need for continued biodiversity research, even in small, well-studied and highly developed countries such as Singapore that can still harbor undescribed biodiversity.

## 2. Materials and Methods

### 2.1. Study Site and Field Sampling

**Figure 1:**
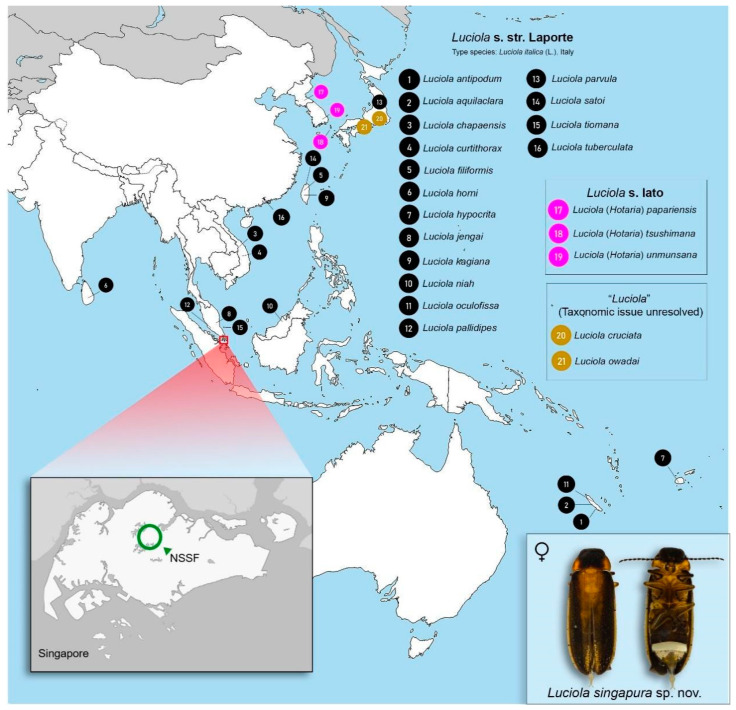
Map of Singapore showing the location of *Luciola singapura* Jusoh & Ballantyne sp. nov. in the Nee Soon Swamp Forest (NSSF) (inset). Numbered dots represent type localities of *Luciola* species in Asia and the Pacific islands that were examined and/or analysed in this study. List of species in Table S1.

Singapore is the second smallest country in Asia (~728 km^2^) and comprises four legally protected nature reserves covering approximately 4% of its total land area [23]. For this study, specimens were collected exclusively from the Nee Soon Swamp Forest (hereafter referred to as NSSF) which is part of the Central Catchment Nature Reserve (Figure 1). The NSSF is an important conservation site that is rich in biodiversity and is the only remaining patch of primary freshwater swamp forest in Singapore [26].

We conducted night surveys in NSSF between 7.30 p.m. and 10.30 p.m. on 9 October 2018, 11 October 2018, 11 January 2019, and 18 January 2019. These surveys specifically targeted specimens that matched the description of *Luciola* sp. 2 *sensu* [24]. In total, four specimens comprising three males and one female were collected, all of which were found during surveys conducted in January. We also examined specimens deposited in the Zoological Reference Collection (ZRC) at the Lee Kong Chian Natural History Museum, Singapore, including two specimens collected by Chan and colleagues in 2009. The ZRC is the sole depository of all specimens examined, including type material.

### 2.2. Morphological Analysis

We conducted morphological examinations using an Olympus SZ51 (Olympus Corporation, Tokyo, Japan) microscope and basic dissection materials. Characters assessed, abbreviations, and dissection of abdominal features follow [3] and are summarized in Table 1. We also included references to diagrams in previously published work where appropriate. Diagnostic morphological characters were checked and compared against 16 published type specimens of *Luciola* from the SE Asian and Australia-Pacific region (Figure 1; Table S1). We imaged specimens using the Dun Inc. Passport II Photomicrography imaging system (with 65 mm MPE Canon Lens) (Dun, Inc., Virginia, USA), and image post-processing was done in Adobe Lightroom (Adobe Inc., California, USA) and stacked in Zerene Stacker (Zerene Systems LLC, Washington, DC, USA).

### 2.3. DNA Extraction, Sequencing and Bioinformatics

For this study, we extracted genomic DNA (gDNA) from three firefly specimens from Singapore (*Pyrocoelia fumigata* Gorham, *Pteroptyx valida* Olivier and *Luciola* sp. 2; see Table S2) using the QIAamp 96 DNA QIAcube HT Kit (QIAGEN, Germany) on the QIAcube platform following an overnight digest of the entire specimen according to the manufacturer’s instructions. The gDNA of firefly samples were then pooled with other species (non fireflies) across three libraries, with each library consisting of DNA from ~100 species and sequenced on an Illumina Hiseq 2500 250-bp paired-end lane (Illumina, San Diego, USA) following library preparation with a NEBNext Ultra™ II DNA Library Prep Kit (NEB, Ipswich, USA) at the Genomic Institute of Singapore (Singapore). A total of 340 million reads were generated from the total run, with each library receiving an average of 113 million reads. Exact coverage is difficult to determine because of the pooling of multiple species in the same library. However, from our estimation, a total of 17.5 million reads were obtained from ~300 species, which gives an average 3.7× coverage for the mitogenomes. After sequencing, Trimmomatic v.0.39 [27] was used for adapter trimming and a BLAST search was conducted on the reads against a reference database of full Lampyridae mitochondrial genomes from NCBI GenBank (see Data S1) to filter out non-mitochondrial reads. The filtered reads were then assembled using four different assemblers: SPAdes v.3.13 [28] (--meta, -k 21,33,55,77), Ray v.2.1.1 [29] (-k 61, -minimumseedlength 100 -minimumcontiglength 1000), IDBA-UD [30] (--mink 60 --maxk 150) and CLCGenomicsWorkbench v.8.0.3. For IDBA-UD, we performed additional quality filtering using PRINSEQ [31] before assembly. The assembled contigs were then merged into supercontigs in Geneious R11 [32] via the De Novo Assemble function (minimum overlap: 1000, 1% maximum mismatches per read). We annotated the mitochondrial genomes with Geneious’ Live Annotate and Predict function using annotated lampyrid mitogenomes from NCBI GenBank and extracted the coding genes as well as rRNA sequences.

### 2.4. Genetic Data and Phylogenetic Analyses

In addition to the mitogenomes sequenced in this study, we also obtained published mitogenomes from eight closely related genera within the subfamily Luciolinae (*Abscondita, Aquatica, Asymmetricata, Curtos, Inflata, Pteroptyx, Pygoluciola* and *Sclerotia*) and partial mitochondrial fragments of *Cytochrome c oxidase subunit 1* (*COX1*) and *Cytochrome c oxidase subunit 2* (*COX2*) from 34 species to fill-in sampling gaps and increase taxonomic coverage. The mitogenomes of *Pyrocoelia rufa* and *P. fumigata* from the subfamily Lampyrinae were used as outgroups. All sequences used in this study and their associated GenBank accession numbers are presented in Table S2.

Sequences were aligned separately using MAFFT v7 [33] and subsequently concatenated using AMAS [34]. Pairwise uncorrected *p*-distances of the *COX1* gene were calculated using MEGA-X [35]. The “complete deletion” option was used for missing data, while other options were left at default settings. We then performed phylogenetic analyses of concatenated data using maximum likelihood (ML) and Bayesian Inference (BI). The ML analysis was implemented in the program IQ-TREE v2.1.1 [36] (partitioned by marker). The best-fit substitution model for each partition was determined using ModelFinder [37] and a 50% majority-rule consensus tree was constructed from 1000 ultrafast bootstrap replicates [38]. To parse out the effects of missing data, we also performed an IQ-TREE analysis on *COX1* sequences only. The BI phylogeny was estimated using BEAST2 [39], implemented through the CIPRES portal [40]. Substitution models for each partition were averaged using bModelTest [41] and the relaxed log-normal and Yule models were used as the clock and tree priors respectively. Two independent Markov chain Monte Carlo (MCMC) chains were executed at 20,000,000 generations each and assessed for convergence using Tracer [42]. A maximum clade credibility tree was obtained from the converged and combined posterior distributions after discarding the first 10% of sampled trees as burn-in.

## 3. Results

### 3.1. Morphology

*Luciola* sp. 2 has a distinctive pale-colored pronotum with median dark marking; a character that is shared with several *Luciola* species. However, it can be distinguished from (a) *Luciola curtithorax* Pic by the ML length to LL; (b) *L. filiformis* Olivier and *L. parvula* by the absence of hind wings or brachelytral in females; (c) *L. horni* by apices of LL narrowed and strongly divergent and anterolateral arms longer than abdominal tergite 8 (T8) entire posterior portion, the posterior margin of which is rounded [3] (p. 92, Figure 268). *Luciola* sp. 2 also differs from the following ten *Luciola* s. str. species in (a) biogeographic distribution—*L. italica* is a European species and the following four species are known only from the Pacific islands: *L. antipodum*, *L. aquilaclara*, *L. hypocrita* and *L. oculofissa* (Figure 1); (b) distinctive ecological state—two species from East Asia: *L. satoi* occurs at high elevations (500–1600 m a.s.l.) in Taiwan [43] (p. 255) and *L. tuberculata* Yiu confined to specific mature woodlands and currently classified as endemic to Hong Kong [44]; (c) body length: *Luciola* sp. 2 is at least twice as small as *L. chapaensis*, *L. jengai* and *L. kagiana* (Table S1). 

Among SE Asian *Luciola* species, *L.* sp. 2 can be distinguished by having a pronotum with a median dark marking and in particular from *L. niah* by not having pale dorsal coloration with black elytral apices [3] (p. 95, Figures 284–287); the separation of aedeagal LL along their inner dorsal length and the ML length in comparison with LL [3] (p. 95, Figures 288–291 Figure 288 Figure 289 Figure 290 Figure 291); *L. pallidipes* by the non-uniform dorsal coloration [3] (p. 98, Figure 296); smaller interocular distance [3] (p. 98, Figure 301) and median anterior margin of aedeagal sheath tergite not apically squarely truncated [3] (p. 99, Figure 305); *L. tiomana* by the posterolateral corners of the pronotum being obtusely rounded [3] (p. 101, Figure 301), median posterior margin of T8 slightly emarginated and median anterior margin of aedeagal sheath tergite prolonged and apically truncated [3] (p. 101, Figure 314). 

### 3.2. Genetic Distance, Phylogenetic Analyses, and Divergence Times

**Figure 2:**
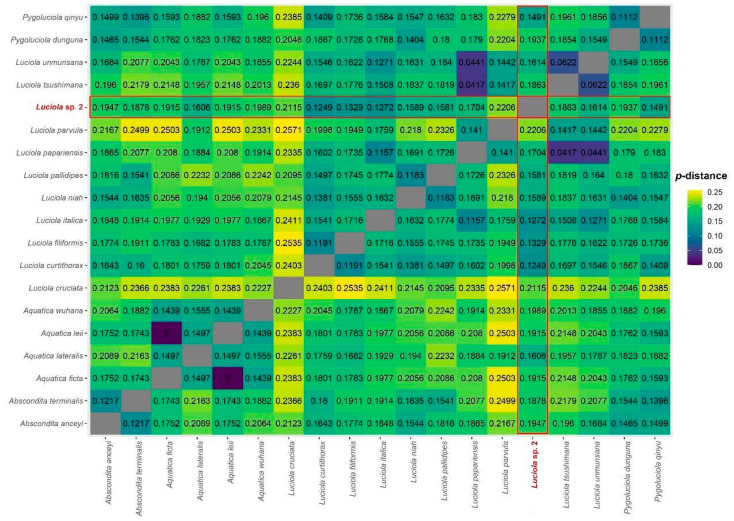
Heatmap of uncorrected *p*-distances among *Luciola* sp. 2 and closely-related taxa based on *COX1*.

A total of 15 mitochondrial markers were sequenced (*12S*, *16S*, *ATP6*, *ATP8*, *COX1*, *COX2*, *COX3*, *Cytochrome*-*b*, *ND1*, *ND2*, *ND3*, *ND4*, *ND4L*, *ND5,* and *ND6*) and the final concatenated sequence matrix consisted of 13,602 base pairs. The uncorrected *p*-distances (based on *COX1*) among *Luciola* sp. 2 and closely-related taxa were consistent with interspecific distances among other lucioline taxa (12.5–22.0%), while *p*-distances among *L. umunsana*, *L. tsushimana* and *L. papariensis* were markedly lower (4.4–6.2%). Distances between *Aquatica leii* and *A. ficta* were 0%, indicating that these taxa could be conspecific (Figure 2).

**Figure 3:**
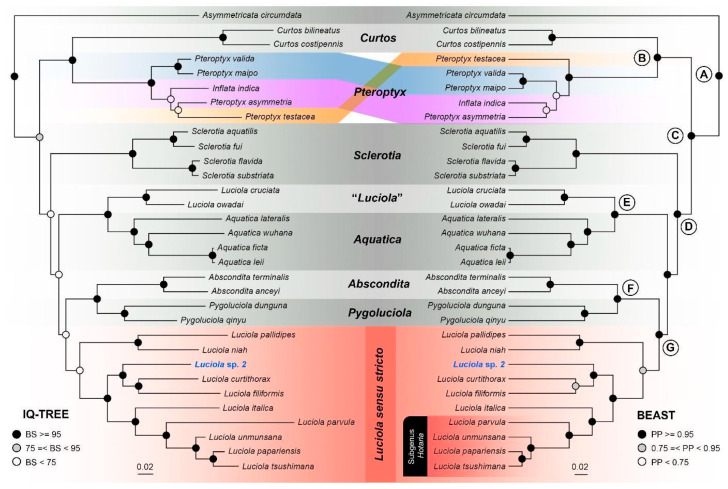
Maximum likelihood (left) and Bayesian (right) phylogenies based on 13,602 bps of mitogenomic data.

Both ML and BI topologies inferred largely similar topologies except for minor differences within the *Pteroptyx/Inflata* clade, which also had weak support in both phylogenies (Figure 3). Despite similar topologies, branch support along the backbone of the BI phylogeny was significantly stronger compared to the ML phylogeny. The genus *Luciola* was paraphyletic with strong support—*L. owadai* and *L. cruciata* from Japan were recovered as the sister lineage to the genus *Aquatica*, while other *Luciola* species formed a clade that was sister to the *Pygoluciola* + *Abscondita* clade. The latter *Luciola* clade corresponds to *Luciola* s. str. proposed by Ballantyne and Lambkin [8]. The genus *Pteroptyx* was also paraphyletic with regard to the genus *Inflata. Luciola* sp. 2 formed a clade with *L. curtithorax* and *L. filiformis* with high support in both phylogenies. The ML phylogeny based purely on *COX1* sequences was weakly supported and produced different topologies with regard to the genera *Sclerotia*, *Pteroptyx*, *Curtos*, and *Asymmetricata*, while relationships among *Aquatica*, *Abscondita*, *Pygoluciola*, and *Luciola* remained the same (albeit with weaker support). *Luciola cruciata* was also recovered as the sister lineage to the *Aquatica* clade with strong support. Similarly, *L*. sp. 2 was also recovered as the sister lineage to *L. filiformis + L. curtithorax.*

### 3.3. Systematics

Our results demonstrate that *Luciola* sp. 2 represents a distinct evolutionary lineage that is genetically and morphologically distinct from other congeners and is thus described as a new species below:

*Luciola singapura* Jusoh and Ballantyne sp. nov.
LSID: urn:lsid:zoobank.org:act:A963B320-8889-4FEC-B152-21E3EFAB76F9
English name: Singapore firefly
Malay name: Kunang-kunang Singapura

#### 3.3.1. Type Material

Holotype, male, in ethanol, with genitalia in a separate vial. Singapore: Nee Soon Swamp Forest, 18 January 2019, 9:00–9:20 p.m., coll. T.W. Wong, S.H. Chan and W.F.A. Jusoh, catalogue no. ZRC_ENT00034035. Paratypes, 1 female, 6 males: All paratypes bear the same locality label as the holotype, i.e., Singapore: Nee Soon Swamp Forest or “Nee Soon Pipeline (Upp Peirce) Freshwater Swamp”; 18 January 2019, 8:30–9:00 p.m., coll. T.W. Wong, S.H. Chan and W.F.A. Jusoh, ZRC_ENT00034034 (1 female, ethanol-preserved specimens); 11 January 2019, 8:30–9:00 p.m., coll. T.W. Wong, S.H. Chan and W.F.A. Jusoh, catalogue no. ZRC_ENT_00034033, sample ID: WFA-2149 (1 male, ethanol-preserved specimen); 22 January 2010, coll. Yeo Suay Hwee and S.H. Chan, ZRC_ENT00034042 (1 male; ethanol-preserved specimen, sample ID: NS010 (sequenced in this study); 22 January 2010, coll. S.H. Chan, 22:15 hrs, catalogue no. ZRC_ENT00034043 (1 male, dry preserved, pinned specimen); 18 January 1990, coll. Y.H. Koo and P.K.L. Ng, catalogue nos. ZRC_ENT_00000010, ZRC_ENT00034044 (2 males, dry preserved specimen, card-mounted); 28 Dec 1989, coll. Y.H. Khoo and K. Snyder, catalogue no. ZRC_ENT00034045 (1 male, dry preserved specimen, card-mounted). 

#### 3.3.2. Diagnosis

A small species (less than 5 mm long) with distinctive dorsal coloration of black head, orange to yellowish brown with dark markings on pronotum, and orange to yellowish brown elytra which have diffuse darker brown to black markings towards the apex, with paler basal markings restricted to basal area near suture and an accumulation of whitish fat body along apex of suture and round elytral apex. Metasternum with median dark marking. 

#### 3.3.3. Description

**Figure 4:**
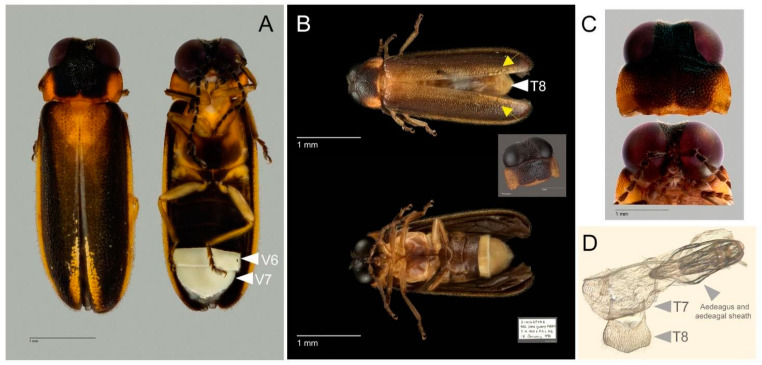
Males of *Luciola singapura* sp. nov. Habitus (**A**,**B**), head and pronotum (**C**); Tergite 8 after dissection (**D**). (**A**). Holotype male, from recent collection preserved in ethanol, light organ (LO) located on ventrite 6 (V6) and ventrite 7 (V7), presence of fat body along apex of suture, arrowed; (**B**). Habitus of paratype male. Coloration of paratype male collected 30 years ago preserved as dry specimen, arrow indicated presence of fat body along suture, tergite 8 (T8) from dorsal view and pronotum (inset); (**C**). Top: Head and pronotum of paratype male, below: Mouthpart and some parts of antenna; (**D**). Dissection parts of holotype male: tergites 7-8 indicated with aedeagus and aedeagal sheath.

Males. 4.44–4.71 mm long. Color (Figure 4A,B): dorsal coloration not concolorous (note that type specimens that were recently collected and preserved in 70% ethanol slightly differ in color compared to dry preserved specimens); pronotum orange to yellowish brown (either orange, Figure 4A or yellowish brown Figure 4B) with median darker markings; mesonotal plates (MN), mesoscutellum (MS) and elytra orange to yellowish brown with elytral apices black or dark brown; elytra semitransparent and underlying hind wings may confuse interpretation of color; head black, antennae and palpi dark brown to black; venter of thorax yellow (or yellow orange) with median dark brown to black marking which reaches the anterior but not the posterior pronotal margin, lateral margin orange (Figure 4A, arrowed; Figure 4B, inset), basal abdominal ventrites (except ventrite 6 (V6) and ventrite 7 (V7)) and abdominal tergites dark brown to black; legs yellowish brown with tibiae and tarsi dark brown (legs yellow, tarsi light brown); Light organ (LO) creamy white (Figure 4A). Pronotum (Figure 4B, inset; Figure 4C): slightly convex-sided; lateral margins diverging laterally along most of their length (B > A, C); width subequal to humeral width; antero- and posterolateral corners rounded obtuse. Elytron: subparallel sided with narrow pale margins. Accumulation of fat body along apex of suture and round elytral apex (Figure 4A,B arrowed). Head (Figure 4C): moderately well exposed in front of pronotum; minimal depression between eyes; greatest head width (GHW) 8 X smallest interocular width (SIW); distance between antennal sockets (ASD) much less than antennal socket greatest diameter (ASW) (sockets are almost contiguous). Antennae: longer than GHW but not as long as 2 X GHW; 11 segments, elongate slender, scape longer than antennal flagellar segments (FS) (Figure 4C). Mouthparts: apical labial palpomere laterally flattened with inner edge dentate. Abdomen (Figure 4A,B,D): LO occupying entire area of V6, 7; V7 a little longer than wide, posterior margin acute, no posterolateral projections (PLP), no median posterior projection (MPP); Tergite 8 (Figure 4D) with anterolateral arms elongate slender slightly shorter than entire posterior portion which is rounded and without median emargination. Aedeagal sheath (Figure 4A,B): apical margin of sheath sternite narrowly prolonged and medianly not emarginated and hairy; median anterior margin of sheath tergite prolonged and apically truncated. Aedeagus (Figure 4B): apices of LL narrow; LL about equal to ML, leaf-like, closely approach along median dorsal line. 

**Figure 5:**
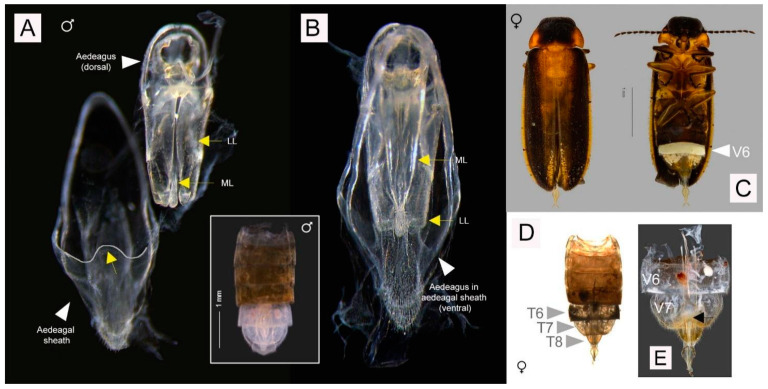
Aedeagal and aedeagal sheath of male (**A**,**B**) and diagnostic characters of female (**C**–**E**) of *L. singapura* sp. nov.; (**A**). Aedeagal sheath, dorsal view, median anterior margin of sheath tergite prolonged and apically truncated, outlined and arrowed; (**B**). Aedeagus, dorsal view and ventral view with arrow indicated median lobe (ML) and lateral lobes (LL); Inset: dorsal view of male abdominal tergites showing aedeagus with aedeagal sheath and tergite 8 still intact; (**C**). Habitus, dorsal and ventral view, LO on V6; (**D**). Dorsal view (after tissue clearing, before dissection), tergites 6-8 indicated; (**E**). Ventral view, dissected part, arrow showing posterior margin of V7 widely emarginated.

Female (Figure 5C–E). 3.93 mm long. Associated by its occurrence in the same habitat within the same time of sampling, similarity of distinctive color patterns and identical DNA barcode with the males. Macropterous and capable of flight. Colored as for male except LO in V6; and V7, 8 are semitransparent, whitish fat reaching anteriorly and to sides but not reaching to posterior margin of V7, posterior margin of V7 widely emarginated (Figure 5C,E, arrowed). Antennae: 11 segmented with elongate scape which is expanded in apical half, pedicel slightly shorter than scape and antennal flagellar segment 1 (FS1), all flagellar segments elongate slender; no flagellomeres expanded. No bursa plate and spermatophore gland observed (Figure 5E).

#### 3.3.4. Etymology

The specific epithet, *singapura*, is the Malay name for the country Singapore, which is the type locality of the new species.

#### 3.3.5. Distribution

Known only from the type locality of NSSF, Central Catchment Nature Reserve in Singapore (see also [24], p. 115, Figure 1) but may also occur in other parts of the island where habitat is suitable. 

#### 3.3.6. Ecology

**Figure 6:**
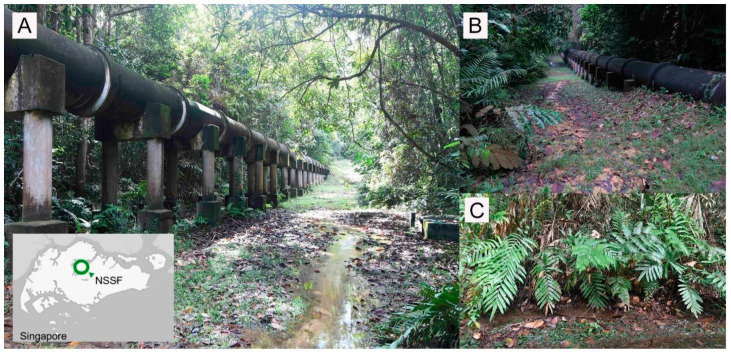
Habitat at Nee Soon Swamp Forest in Singapore (map, inset) where *L. singapura* was found; (**A**,**B**). The freshwater swamp is situated within the water catchment along a water supply pipeline; (**C**). Adult fireflies of *L. singapura* were observed flying around and/or perching among shrubs beneath the pipeline with the majority of them perched on ferns.

The habitat is a swampy area adjacent to the water pipeline system (Figure 6A,B) with dense vegetation, damp leaf litter, and high soil moisture. Chan and colleagues observed the adults congregating within a very small area at a specific location, most of which were perching and flashing on a species of fern identified as *Blechnum finlaysonianum* Wall. (Figure 6C). Some individuals were observed flying low while emitting flickering yellow flashes among shrubs and at the fringe of the forest between 8:00 p.m. and 10:15 p.m. No larvae were spotted.

## 4. Discussion

### 4.1. Phylogenetic Relationships and Evolutionary History

Our analyses strongly supported the paraphyly of *Luciola cruciata* and *L. owadai* that was inferred as the sister lineage to the genus *Aquatica* as opposed to other *Luciola* species. Similar to the genus *Aquatica*, larvae of *L. cruciata* and *L. owadai* are also aquatic (as opposed to larvae of other *Luciola* species which are semi-aquatic or terrestrial) [2,3,14], thereby providing additional support for their phylogenetic affinity with *Aquatica* as opposed to *Luciola*. Our results, which are also consistent with previous studies, unequivocally demonstrate that *L. cruciata* and *L. owadai* do not belong to the genus *Luciola* and should either be subsumed under the genus *Aquatica* or recognized as a new genus. Consequently, we formally remove *L. cruciata* and *L. owadai* from the genus *Luciola* and provisionally consider them as *incertae sedis* (“*Luciola*”) pending future investigation. Henceforth, all references to the genus *Luciola* exclude these two taxa. 

Prior to our mitogenome-based phylogenetic analyses, the subfamily Luciolinae had been subjected to several phylogenetic studies, the most comprehensive of which performed maximum parsimony (MP) and BI analyses of concatenated molecular (25 taxa) and morphological data (158 taxa) [3,15]. In general, the MP analysis produced a topology similar to ours, but the BI analysis produced slightly different relationships. In our analysis, the genus *Inflata* was embedded within *Pteroptyx* (albeit with low support) as opposed to being reciprocally monophyletic with the genus *Pteroptyx* as inferred by [1,15]. *Inflata* is a monotypic genus that closely resembles *Pteroptyx* species in morphology, particularly of Group II (non-deflexed elytra + MFC + entire light organ (ELO), as defined by [45]) except that *Inflata* possesses entire LOs in V7 without the bulbous ML. Due to poor branch support, ambiguous morphological characters, and limited samples of *Pteroptyx* species assessed in this study, the phylogenetic placement and generic status of *Inflata* remain uncertain and would likely require additional molecular data to resolve. Concerning the similarity in DNA sequences of *Aquatica leii* and *A. ficta,* a re-examination of voucher specimens is necessary to determine whether those sequences have been misidentified. 

For *Luciola* species under the subgenus *Hotaria* sensu McDermott, our analyses showed that they form a derived subclade within *Luciola*, thereby supporting their status at the subgenus rank as demonstrated by [22]. The type species of the subgenus *Hotaria*, *L. parvula* forms a clade with three other species, *L. unmunsana, L. papariensis* and *L. tsushimana*, which according to our results, are part of *Luciola* s. str. Morphologically, these three species share many similarities, which makes taxonomic identification at the species level challenging even for experienced taxonomists. Our results were not able to accept or reject the synonymies of *L. papariensis* and *L. tsushimana* with *L. unmunsana* as suggested by [23]. *Luciola unmunsana*, *L. papariensis*, and *L. tsushimana* were moderately divergent from each other (4–6%; see [20,22] for more detailed discussions on this group). To exacerbate matters, the type specimens of *L. unmunsana* and *L. papariensis* are lost [3] (p. 104). We, therefore, refrain from making any taxonomic changes to these three species other than provisionally recognizing them as part of *Luciola* s. str. (see 4.2). 

### 4.2. Updated Notes on the Species of Luciola s. str. Laporte throughout the SE Asian and Australopacific Region

With the description of *Luciola singapura* sp. nov., there are now 53 nominal species in the genus *Luciola* throughout the SE Asian and Australopacific region including species from *Luciola* s. str. Laporte and *Luciola* s. lato sensu Ballantyne. We expand the definition of *Luciola* s. str. in this region to comprise 20 species including the new species, *Luciola singapura* sp. nov. The provisional inclusion of *Luciola jengai* (see remarks in [3], p. 96), *L. papariensis, L. tsushimana*, and *L. unmunsana* may change following future taxonomic investigations.

*Luciola* s. str. Laporte:

1. *antipodum* (Bourgeois, 1884) [46]
2. *aquilaclara* Ballantyne, 2013 in [8]
3. *chapaensis* Pic, 1923 [47]
4. *curtithorax* Pic, 1927 [48]
5. *filiformis* Olivier, 1913 [49]
6. *horni* Bourgeois, 1905 [50]
7. *hypocrita* Olivier, 1888 [51]
8. *jengai* Nada, 2019 in [3] (provisional)
9. *kagiana* Matsumura, 1928 [52]
10. *niah* Jusoh, 2019 in [3]
11. *oculofissa* Ballantyne, 2013 in [8]
12. *pallidipes* Pic, 1928 [53]
13. *papariensis* Doi, 1932 [54] (provisional)
14. *parvula* Kiesenwetter, 1874 [55]
15. *satoi* Jeng and Yang, 2003 in [43]
16. *singapura* Jusoh and Ballantyne sp. nov.
17. *tiomana* Ballantyne, 2019 in [3]
18. *tsushimana* Nakane, 1970 [56] (provisional)
19. *tuberculata* Yiu, 2017 [44]
20. *unmunsana* Doi, 1931 [57] (provisional)

### 4.3. Status and Conservation of Fireflies in Singapore

Hitherto, Singapore has 14 species of fireflies, including *Luciola singapura* sp. nov. [24,25,58]. The list also comprises several morphospecies (e.g., *Curtos* sp., *Diaphanes* sp., *Stenocladius* sp.) from previous surveys [24,58] and three type specimens from the non-luminous firefly group deposited in the Muséum national d’Histoire naturelle (MNHN) in Paris (*Ototreta bakeri* Pic, *Lucidina wallacei* Pic and *Flabellotreta inapicalis* Pic) collected from Singapore [25]. The Comtesse’s firefly (*Pteroptyx bearni* Olivier), which is a well-known congregating firefly species in SE Asian mangroves has not been sighted in Singapore since 1909 and is assumed to be extirpated [25]. The following five species have been observed in primary forest, secondary forest, freshwater swamp, mangrove, grassland, and scrubland habitats: *Abscondita pallescens* (Gorham), *Colophotia praeusta* (Eschscholtz), *Pteroptyx malaccae* Gorham, *Pteroptyx valida* Olivier, and *Pyrocoelia fumigata* Gorham, of which only *P. valida* is listed as endangered (EN) in the Singapore Red Data Book [59].

*Luciola singapura* sp. nov. is a rare species that is only known from the type locality, which is a freshwater swamp forest within the Central Catchment Nature Reserve. More studies are needed to determine if this species is a freshwater swamp specialist, or whether it could also occur in other habitats. Although the type locality is legally protected from development and clearing, the long-term protection of this species will require a deeper understanding of its ecology and distribution, both of which are lacking. 

## 5. Conclusions

The discovery of a new species of firefly from the last remaining freshwater swamp forest in Singapore—one of the most developed and urbanized countries in the world—highlights the importance of continued biodiversity research in the region. This study also underscores the utility of molecular approaches to resolve taxonomic problems and better understand the evolutionary history of fireflies.

## Figures and Tables

**Table 1:** Abbreviations and definitions of morphological characters examined in this study.

Character	Definition
a	aedeagal dimension, distance from dorsal base of the lateral lobes to the apex of the median lobe (see b); expressed as b/a [15] (p. 25, Figure 25; p.37, Figure 104)
A	pronotal dimension measured from above; width across anterior third
ASD	distance between antennal sockets
ASW	antennal socket greatest diameter
b	aedeagal dimension, distance from the dorsal base of the lateral lobes to the apex of the lateral lobes (see a); expressed as b/a [15] (p. 25, Figure 25; p.37, Figure 104)
B	pronotal dimension measured from above; width across middle
BL	body length measured as median length of pronotum plus length of elytron
C	pronotal dimension measured from above; width across posterior third
FS	antennal flagellar segments
GHW	greatest head width (across eyes, measured parallel to ASD)
L	length
Legs 1, 2 etc	legs and parts of legs are referred to by their segment number e.g., legs 1 are prothoracic legs; tarsi 2 = mesothoracic tarsi; femora 3 = metathoracic femora.
LL	lateral lobes, aedeagus
LO	light organ
ML	median lobe of the aedeagus
MN	mesonotal plates
MS	mesoscutellum
MPP	median posterior projection ventrite 7 male only
PLP	posterolateral projections ventrite 7 male only
SIW	smallest interocular width (measured horizontally and may be on the same level as ASD, ASW, or above it if the eyes are closer there)
T7, 8 etc.	abdominal tergites
V6, 7 etc.	abdominal ventrites, referred to by actual, not visible number
W	width
W/L	width/length
x	times

## Data Availability

The data presented in this study are available as Supplementary Materials.

## Supplementary Materials

The following are available online at https://www.mdpi.com/2076-2615/11/3/687/s1. Table S1: Diagnostic morphological characters of 17 species of *Luciola* s. str. found in Asia and the Pacific Islands, including *Luciola* sp. 2.; Table S2: List of genetic samples used in this study and their corresponding GenBank accession numbers; Data S1: GI numbers of Lampyridae from NCBI GenBank used for BLAST database; Sequence alignment (Nexus format) and all phylogenetic trees (Newick format).
