# Supplementary material for: Molecular Systematics of the Firefly Genus Luciola (Coleoptera: Lampyridae: Luciolinae) with the Description of a New Species from Singapore
Source: Animals (Basel). 2021 Mar 4;11(3):687. doi: 10.3390/ani11030687 (PMC7998795; doi:10.3390/ani11030687)
Supplement: Supplementary file 1 [file animals-11-00687-s001.zip › Table S1-revised2.pdf]

Table S1: Diagnostic morphological characters of 17 species of *Luciola* **s. str.** found in Asia and the Pacific Islands, including *Luciola* sp. 2. Type species of *Luciola, L. italica* from Italy not included here.

	1	2	3	4	5	6	7	8	9	10	11	12	13	14	15	16	17
**Species name / Character**	***Luciola antipodum* (Bourgeois, 1884)**	***Luciola aquilaclara* Ballantyne, 2013**	***Luciola chapaensis* Pic, 1923**	***Luciola curtithorax* Pic, 1927**	***Luciola filiformis* Olivier, 1913**	***Luciola horni* Bourgeois, 1905**	***Luciola hypocrita* Olivier, 1888**	***Luciola jengai* Nada, 2019 in Ballantyne et al. 2019**	***Luciola kagiana* Matsumura, 1928**	***Luciola niah* Jusoh, 2019 in Ballantyne et al. 2019**	***Luciola oculofissa* Ballantyne, 2013**	***Luciola pallidipes* Pic, 1928**	***Luciola parvula* Kiesenwetter, 1874**	***Luciola satoi* Jeng & Yang, 2003 in Jeng et al. 2003**	***Luciola tiomana* Ballantyne, 2019 in Ballantyne et al. 2019**	***Luciola tuberculata* Yiu, 2017**	***Luciola* sp. 2 (this study)**
Measurement: BL body length taken as median length of pronotum plus length of elytron (in mm)	3.6–4.7	4.1–6.0	10	6.5–8.0	5	6	8.4–8.9	8.7	10.5−15	4.8–6.1	3.6–4.5	6	6−9	6.5−10	4.2–4.3	7.3–7.8	4.44–4.71
Measurement: EL elytron length (in mm)	3.1–3.7	3.3–4.8	not mentioned	not mentioned	not mentioned	not mentioned	not mentioned	not mentioned	not mentioned	not mentioned	2.8–3.5	3.55	not mentioned	not mentioned	3.47	6−7	4.07
Measurement: W/L pronotum width/length	1.4–1.5	1.3–1.5	wider than long	not mentioned	not mentioned	not mentioned	1.5	not mentioned	not mentioned	not mentioned	1.2–1.5	2	not mentioned	not mentioned	1.6	1.7-1.9 (L/W)	2.0
Measurement: GHW in comparison with SIW (average)	11 x SIW	10 x SIW	10 x SIW	10 x SIW(a)	not mentioned	6 x SIW	7 x SIW	10 X SIW	not mentioned	11 x SIW	12 x SIW	3 x SIW	not mentioned	not mentioned	10 x SIW	not mentioned	7 x SIW
Measurement: GHW (if available)	0.9–1.3	1.1–1.4	not mentioned	1.53(a)	not mentioned	not mentioned	1.8–2.0	not mentioned	not mentioned	not mentioned	1.2	1	not mentioned	not mentioned	1.21	not mentioned	1.22
Measurement: antennae length in comparison with GHW	not mentioned	not mentioned	2 x GHW	length>GHW and < twice GHW(a)	not mentioned	(incomplete)	length>GHW and < twice GHW	not mentioned	not mentioned	length>GHW and < twice GHW	about the same or slightly shorter than GHW)	2.5 x GHW	not mentioned	not mentioned	length>GHW and < twice GHW	not mentioned	length>GHW and < twice GHW
Measurement: comparison between ASD and ASW	ASD < ASW	ASD < ASW	ASD < ASW	possibly, ASD < ASW	not mentioned	ASD < ASW	ASD < ASW	ASD < ASW	not mentioned	ASD < ASW	ASD < ASW	ASD < ASW	not mentioned	not mentioned	ASD < ASW	not mentioned	ASD < ASW
Measurement: SIW (if available)	0.1	0.1–0.15	not mentioned	0.16(a)	not mentioned	not mentioned	0.2–0.3	not mentioned	not mentioned	not mentioned	0.1	0.3	not mentioned	not mentioned	0.12	not mentioned	0.14
Light organs in at least one abdominal ventrite	present (in V6 and V7)	present (in V6 and V7)	present (in V6 and V7)	present (in V6 and V7)	present (in V6 and V7)	present (in V6 and V7)	present (in V6 only)	present (in V6 and V7)	present (in V6 and V7)	present (in V6 and V7)	absent	present (in V6 and V7)	present (in V6 and V7)	present (in V6 and V7)	present (in V6 and V7)	present (in V6 and V7)	present (in V6 and V7)
Eyes with strong posterolateral emargination clearly visible in resting head	present	absent	absent	absent	absent	absent	present	absent	absent	absent	present	absent	absent	absent	absent	absent	absent
Colour: dorsal colouration male (colour)	concolorous (deep gray)	concolorous (deep gray)	not concolourous	not concolourous	not concolourous	not concolourous	concolorous (deep gray)	pale yellow with small dark brown markings at elytral apex	not concolourous	yellowish brown with black elytral apices	concolorous (deep gray)	concolourous (black)	not concolourous	not concolourous	not concolourous	not concolourous	not concolourous
Colour: pronotum male	unformly dark brown coloured	deep gray to black grey and often contrasting with the slightly paler elytra	pale coloured (yellow to brown) with a median dark marking	pale coloured (yellow to brown) with a median dark marking	pale coloured (reddish or yellow) with a median dark marking which reaches the anterior but not the posterior pronotal margin	pale coloured (yellow to brown) with a median dark marking	uniformly deep gray/ black	uniformly light cream coloured with no dark markings	uniformly coloured (pinkish) with no dark markings	uniformly ligt brown coloured with no dark markings	unformly black coloured	black	pale coloured (yellow to brown) with a median dark marking	uniformly coloured (pinkish) with no dark markings	dark colour with narrow brownish orange band across posterior margin	pale coloured (yellow to brown) with a median dark marking and a pair of longitudinal protuberances at sub-basal region	pale coloured (orange/ yellowish brown) with a median dark marking which reaches the anterior but not the posterior pronotal margin, lateral margin orange
Colour: underside of body (venter)	pale brown	brown	brownish	dark brown(b)	not mentioned	pale coloured (yellow to brown) with median darker markings on ventrites anterior to LO	pale brown	orange yellow but V4 and V5 with median dark brown markings	black	*yellowish brown*	pale brown except for black head	dark brown to black	not mentioned	dark brown metasternum	very dark brown to black	most parts light brown; except apical end of mandibles, maxillary palpi, labial palpi, sometimes median region of metasternum, interior side of tibiae, tarsi, V3 & V4 (except lateral sides), V5 dark brown to black	orange (fresh specimen) to yellowish brown (dry) with a median dark markings
Colour: MN; MS	lighter brown	not mentioned	brown	MS orange(b)	black MS	paler yellow	MN slightly paler grey and semitransparent;MS shiny deep grey almost black	MN creamy white; MS semitransparent and pale yellow	MS yellowish brown	MN, MS yellowish brown	MN, MS dark brown	MN & MS black	not mentioned	not explicitly mentioned but "prothorax rose coloured (pinkish red) except for central apex dark brown and margins yellowish brown"	MN and MS black (holotype, dry preserved specimen), brownish orange (wet preserved specimen) in paratypes	MS orange	MN and MS orange (live or freshly caught specimen) to yellowish brown (dry preserved specimen)
Pronotum: shape	not mentioned	not mentioned	subparallel-sided	transverse; pronotum wider than humeral width and wider than long	not mentioned	subparallel sided	close to sub-parallel-sided	not determined	not mentioned	slightly wider across middle (B > A, C)	not mentioned	subparallel-sided	not mentioned	subtrapezoidal	subparallel-sided	transverse	slightly convex-sided
Pronotum: anterolateral corner	rounded obtuse	rounded obtuse	rounded obtuse	rounded(b)	not mentioned	rounded	rounded obtuse	rounded obtuse	not mentioned, probably rounded	angulate obtuse	rounded obtuse	angulate obtuse	not mentioned	rounded	rounded obtuse	broadly rounded	rounded obtuse
Pronotum: posterolateral corner	rounded obtuse	rounded obtuse	slightly acute	slightly acute(b)	not mentioned	rounded obtuse	rounded obtuse	acute	not mentioned, probably rounded obtuse	angulate	rounded obtuse	angulate obtuse	not mentioned	rounded	acute	gently projected upward	rounded obtuse
Pronotum: pronounced tubercles along posterior margin	absent	absent	absent	absent(b)	absent	absent	absent	absent	absent	absent	absent	absent	absent	absent	absent	present	absent

Head	visible when head retracted	well exposed in front of pronotum	well exposed in front of pronotum	well exposed in front of pronotum, larger than L filiformis(b)	not mentioned	well exposed in front of pronotum	well exposed in front of pronotum	barely depressed between eyes	well exposed in front of pronotum	visible when head retracted	elongate and protruding beyond anterior margin of pronotum	visible when head retracted	not mentioned	well exposed in front of pronotum	visible when head retracted	well exposed in front of pronotum	moderately well exposed in front of pronotum
Proximity antennal sockets	almost contiguous	almost contiguous	almost contiguous	close but not contiguous(b)	not mentioned	almost contiguous	almost contiguous	close but not contiguous	not mentioned	almost contiguous	contiguous	very close but not contiguous	not mentioned	not determined	almost contiguous	not mentioned	almost contiguous
Elytron: shape	subparallel-sided	subparallel-sided	subparallel-sided	elongate and parallel sided(b)	not mentioned	parallel-sided	subparallel-sided	subparallel-sided	not mentioned, most likely subparallel sided	subparallel-sided	subparallel-sided	subparallel-sided	not mentioned	subparallel-sided	subparallel-sided	elongate and parallel sided	subparallel-sided; narrow pale margins
Elytron: accumulation of fat body along apex of suture and round elytral apex	absent	not mentioned	present (whitish)	no(b)	not mentioned	absent	absent	present	not mentioned, most likely absent	absent	absent	absent	not mentioned	not mentioned	present (whitish)	absent	present (whitish)
Tergite 8: anterolateral arms longer than the posterior entire part of T8	not mentioned	not mentioned	no	no(b)	not mentioned	yes	no	no	no	no	not mentioned	no	not mentioned	not mentioned	no	no	no
Aedeagal sheath: median anterior margin of sheath tergite	not mentioned	not mentioned	slightly prolonged	produced and rounded	widely prolonged and apically rounded	Mid anterior margin aedeagal sheath tergite prolonged and apically truncated	not mentioned	not produced	not produced	strongly prolonged and apically pointed	not mentioned	broadly produced and apically squarely truncate	not mentioned	not mentioned	slightly produced forward, not apically truncated	not mentioned, probably not produced	prolonged and apically truncated
Aedeagus: separation of LL along their inner dorsal length	yes	yes	LL, leaf-like, closely approach along median dorsal line with apices of LL expanded	LL, elongate slender, leaf-like, closely approach along median dorsal line with apices of LL expanded (b)	LL, closely approach along median dorsal line with apices of LL expanded (c)	apices of LL narrowed and strongly divergent	yes	LL, not leaf-like, parallel sided with inner dorsal margins close and slightly divergent posteriorly	LL, leaf-like, closely approach along median dorsal line with apices of LL expanded	LL widely divergent	yes	LL, leaf-like, closely approach along median dorsal line with apices of LL expanded	not mentioned	LL, leaf-like, closely approach along median dorsal line with apices of LL expanded	not mentioned	LL, leaf-like, closely approach along median dorsal line with apices of LL expanded	LL, leaf-like, closely approach along median dorsal line
ML length compared to LL	shorter than LL	shorter than LL	slightly longer than LL	shorter than LL (b)	about equal to LL(c)	shorter than LL	no	slightly longer than LL	slightly longer than LL	shorter than LL	shorter than LL	shorter than LL	not mentioned	about equal to LL	slightly longer than LL	slightly shorter than LL	about equal to LL
Female: wings development	unknown	unknown, probably flightless	unknown	macropterous, coloured as male and presumed capable of flight	brachelytral	Unknown	flightless	Macropterous. Not reliably associated but based on the similarity of abdominal colour pattern	macropterous, coloured as male and presumed capable of flight	Macropterous and capable of flight; associated with male by DNA sequence	unknown, probably flightless	Female reliably associated by DNA sequence (in this study), coloured as for male and macropterous	flightless, no hind wing	macropterous	unknown	macropterous, only occasionally flies	macropterous, coloured as male and capable of flight. Associated with male by DNA sequence
Distribution (Region: Geographic Unit)	Australopacific: Melanesia	Australopacific: Melanesia	SE Asia	East and SE Asia	East Asia	South Asia	Australopacific: Melanesia	SE Asia	East Asia	SE Asia	Australopacific: Melanesia	SE Asia	East Asia	East Asia	SE Asia	East Asia	SE Asia
Type locality	New Caledonia (Kanala)	New Caledonia	Vietnam ('Tonkin')	Vietnam (Hoa-Binh)	Taiwan (Formosa)	Sri Lanka	Fiji	Pahang (Fraser's Hill); Terengganu (Jengai)	Taiwan (Formosa)	Sarawak (Lambir, Niah, Kapit)	New Caledonia	Peninsular Malaysia (Malacca); India (Sikkim)	Japan	Taiwan (Sumakusu)	Johor (Tioman island) and Terengganu	Hong Kong	Singapore
Habitat	Unknown	North to south in New Caledonia and at altitudes both above and below 300m	Possibly highlands based on materials examined from other localities e.g.South Annam, 3000 feet (914 above sea level), Cameron Highlands and Banjaran Titiwangsa	Observed to be common in Taiwan (below 1,500 m)	probably highlands at certain altitudes	Unknown	Other materials examined were found in plantation forest, 150m litter and moss	Low dipterocarp forest	Unknown	Low dipterocarp forest	In southern part of New Caledonia the species was found at elevations lower than 300m	possibly highland forest (200–2400 m above sea level) based on materials examined from other localities e.g. Fraser Hill, Gunung Nuang and Mt. Tambuyukon, Sabah	Unknown	higher than 500 m and up to 1600 m above sea level	Low dipterocarp forest, 10–600 m above sea level	Not explicitly mentioned. Found in Ng Tung Chai Valley. Presumed endemic to Hong Kong	Freshwater swamp

Footnotes:
(a) measured from photo of *L. curtithorax* of Tai Po Kau
(b) examined from photo of *L. curtithorax* of Hong Kong
(c) on the basis of illustration of *L. filiformis* in Jeng et al. 2003
