# Supplementary material for: Molecular Systematics of the Firefly Genus Luciola (Coleoptera: Lampyridae: Luciolinae) with the Description of a New Species from Singapore
Source: Animals (Basel). 2021 Mar 4;11(3):687. doi: 10.3390/ani11030687 (PMC7998795; doi:10.3390/ani11030687)
Supplement: Supplementary file 1 [file animals-11-00687-s001.zip › Table S2-revised2.pdf]

Supplemental Table S2: List of genetic samples used in this study and their corresponding GenBank accession numbers.

**Currently accepted name**	**12S**	**16S**	**ATP6**	**ATP8**	**COX1**	**COX2**	**COX3**	**CYTB**	**ND1**	**ND2**	**ND3**	**ND4**	**ND4L**	**ND5**	**ND6**
Abscondita anceyi	NC039706	NC039706	NC039706	NC039706	NC039706	NC039706	NC039706	NC039706	NC039706	NC039706	NC039706	NC039706	NC039706	NC039706	NC039706
Abscondita terminalis	NC044776	NC044776	NC044776	NC044776	NC044776	NC044776	NC044776	NC044776	NC044776	NC044776	NC044776	NC044776	NC044776	NC044776	NC044776
Aquatica ficta	NC035060	NC035060	NC035060	NC035060	NC035060	NC035060	NC035060	NC035060	NC035060	NC035060	NC035060	NC035060	NC035060	NC035060	NC035060
Aquatica lateralis	LC306678	LC306678	LC306678	LC306678	LC306678	LC306678	LC306678	LC306678	LC306678	LC306678	LC306678	LC306678	LC306678	LC306678	LC306678
Aquatica leii	NC025276	NC025276	NC025276	NC025276	NC025276	NC025276	NC025276	NC025276	NC025276	NC025276	NC025276	NC025276	NC025276	NC025276	NC025276
Aquatica wuhana	NC035061	NC035061	NC035061	NC035061	NC035061	NC035061	NC035061	NC035061	NC035061	NC035061	NC035061	NC035061	NC035061	NC035061	NC035061
Asymmetricata circumdata	NC032062	NC032062	NC032062	NC032062	NC032062	NC032062	NC032062	NC032062	NC032062	NC032062	NC032062	NC032062	NC032062	NC032062	NC032062
Curtos bilineatus	NC044789	NC044789	NC044789	NC044789	NC044789	NC044789	NC044789	NC044789	NC044789	NC044789	NC044789	NC044789	NC044789	NC044789	NC044789
Curtos costipennis	MK609965	MK609965	MK609965	MK609965	MK609965	MK609965	MK609965	MK609965	MK609965	MK609965	MK609965	MK609965	MK609965	MK609965	MK609965
Inflata indica	MH427718	MH427718	MH427718	MH427718	MH427718	MH427718	MH427718	MH427718	MH427718	MH427718	MH427718	MH427718	MH427718	MH427718	MH427718
Luciola cruciata	LC306677	LC306677	LC306677	LC306677	LC306677	LC306677	LC306677	LC306677	LC306677	LC306677	LC306677	LC306677	LC306677	LC306677	LC306677
Luciola curtithorax	NC038225	NC038225	NC038225	NC038225	NC038225	NC038225	NC038225	NC038225	NC038225	NC038225	NC038225	NC038225	NC038225	NC038225	NC038225
Luciola filiformis (=yayeyamana)	--	--	--	--	AB608761	--	--	--	--	--	--	--	--	--	--
Luciola italica	--	--	--	--	KM448734	--	--	--	--	--	--	--	--	--	--
Luciola niah	--	--	--	--	KY572917	--	--	--	--	--	--	--	--	--	--
Luciola owadai	--	--	--	--	--	AB051196	--	--	--	--	--	--	--	--	--
Luciola papariensis	--	--	--	--	MK778979	--	--	--	--	--	--	--	--	--	--
Luciola parvula	--	--	--	--	AB608763	--	--	--	--	--	--	--	--	--	--
***Luciola* sp. 2 (*Luciola singapura* sp. nov.)**	MW620428	MW620429	MW620430	MW620431	MW620432	MW620433	MW620434	MW620435	MW620436	MW620437	MW620438	MW620439	MW620440	MW620441	MW620442
Luciola tsushimana	--	--	--	--	AF485358	--	--	--	--	--	--	--	--	--	--
Luciola unmunsana	MT134039	MT134039	MT134039	MT134039	MT134039	MT134039	MT134039	MT134039	MT134039	MT134039	MT134039	MT134039	MT134039	MT134039	MT134039
Luciola pallidipes	--	--	--	--	MW602289	--	--	--	--	--	--	--	--	--	--
Pteroptyx asymmetria	--	--	--	--	KY572920	--	--	--	--	--	--	--	--	--	--
Pteroptyx maipo	NC036353	NC036353	NC036353	NC036353	NC036353	NC036353	NC036353	NC036353	NC036353	NC036353	NC036353	NC036353	NC036353	NC036353	NC036353
Pteroptyx testacea	--	--	--	--	KY572919	--	--	--	--	--	--	--	--	--	--
Pteroptyx valida	MW620443	MW620444	MW620445	MW620446	MW620447	MW620448	MW620449	MW620450	MW620451	MW620452	MW620453	MW620454	MW620455	MW620456	MW620457
Pygoluciola dunguna	--	--	--	--	MT106239	--	--	--	--	--	--	--	--	--	--
Pygoluciola qinyu	MK292093	MK292093	MK292093	MK292093	MK292093	MK292093	MK292093	MK292093	MK292093	MK292093	MK292093	MK292093	MK292093	MK292093	MK292093
Sclerotia aquatilis	--	--	--	--	KP763466	--	--	--	--	--	--	--	--	--	--
Sclerotia flavida	--	--	--	--	KP763461	--	--	--	--	--	--	--	--	--	--
Sclerotia fui	--	--	--	--	KP763464	--	--	--	--	--	--	--	--	--	--
Sclerotia substriata	KP313820	KP313820	KP313820	KP313820	KP313820	KP313820	KP313820	KP313820	KP313820	KP313820	KP313820	KP313820	KP313820	KP313820	KP313820
Pyrocoelia rufa	AF452048	AF452048	AF452048	AF452048	AF452048	AF452048	AF452048	AF452048	AF452048	AF452048	AF452048	AF452048	AF452048	AF452048	AF452048
Pyrocoelia fumigata	MW620458	MW620459	MW620460	MW620461	MW620462	MW620463	MW620464	MW620465	MW620466	MW620467	MW620468	MW620469	MW620470	MW620471	MW620472
